# Case Report: A case of primary renal synovial sarcoma

**DOI:** 10.3389/fsurg.2026.1843112

**Published:** 2026-05-21

**Authors:** Xuan Li, Jiahao Su, Zijie Tang, Yuan Li, Xiaogang Feng, Feng Lin, Hanyu Chen, Qizhong Fu, Xujie Liu, Ying Liu

**Affiliations:** 1 Department of Urology Surgery, Affiliated Zhongshan Hospital of Dalian University, Dalian, China; 2 College of Basic Medical Sciences, Dalian Medical University, Dalian, China

**Keywords:** Bcl-2, ifosfamide, kidney neoplasms, SS18-SSX2, synovial sarcoma

## Abstract

This case report describes a 53-year-old male patient with primary renal synovial sarcoma. He initially presented with intermittent pain in the lumbar and abdominal regions. Imaging revealed a left renal mass measuring approximately 13.3 cm × 11.6 cm. After radical left nephrectomy, pathological diagnosis was confirmed through immunohistochemical analysis and genetic testing, which identified an SS18-SSX2 fusion, leading to a definitive diagnosis of primary renal synovial sarcoma. Postoperatively, the patient received chemotherapy with epirubicin combined with ifosfamide. Follow-up showed no significant evidence of metastasis or recurrence. This report aims to provide clinical reference regarding the imaging findings and diagnosis of this rare disease, primary renal synovial sarcoma.

## 1 Introduction

Synovial sarcoma is a relatively rare and poorly differentiated soft tissue sarcoma that commonly occurs in the joints of the limbs, especially the lower limbs (1). Primary renal synovial sarcoma is even rarer, accounting for less than 2% of all renal malignancies (2). Its clinical manifestations and imaging features are not significantly different from those of common renal cell carcinoma, and further diagnosis requires a combination of postoperative immunohistochemistry and genetic analysis. We report a case of primary renal synovial sarcoma. The patient, a 53-year-old male, initially presented with intermittent left flank and abdominal pain. He does not have obvious symptoms of hematuria. Subsequent imaging studies, including computed tomography (CT) and magnetic resonance imaging (MRI), revealed a large, heterogeneously enhancing left renal mass measuring approximately 13.3 cm × 11.6 cm, with both modalities suggesting a malignant lesion. A radical left nephrectomy was subsequently performed. Postoperative pathological examination with immunohistochemical (IHC) staining was suggestive of synovial sarcoma. To confirm the diagnosis, further genetic analysis was conducted, which identified the presence of the SS18-SSX2 fusion gene, thereby confirming the diagnosis of synovial sarcoma. The patient received four cycles of postoperative chemotherapy, and no recurrence or metastasis was found during follow-up. This case report comprehensively details the diagnostic workup, therapeutic intervention, and follow-up course for this patient, aiming to provide clinical reference for the management of this rare entity, primary renal synovial sarcoma.

## 2 Case presentation

**Figure 1:**
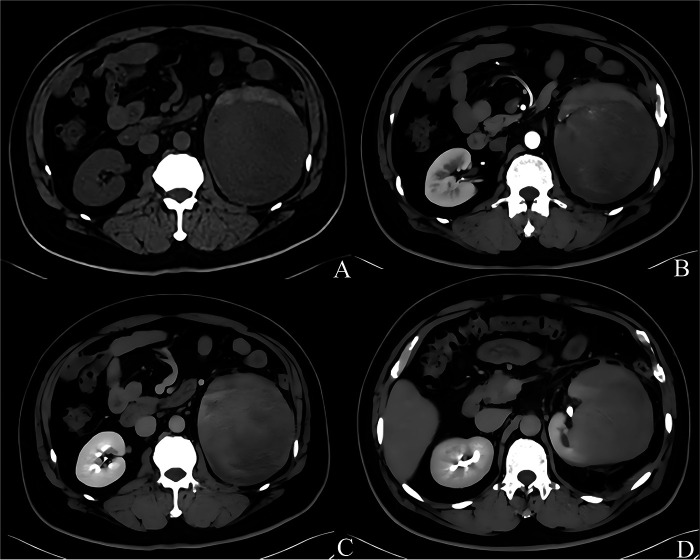
CT of both kidneys [panels **(A–D)** correspond to the unenhanced phase, arterial phase, venous phase, and delayed phase, respectively].

**Figure 2:**
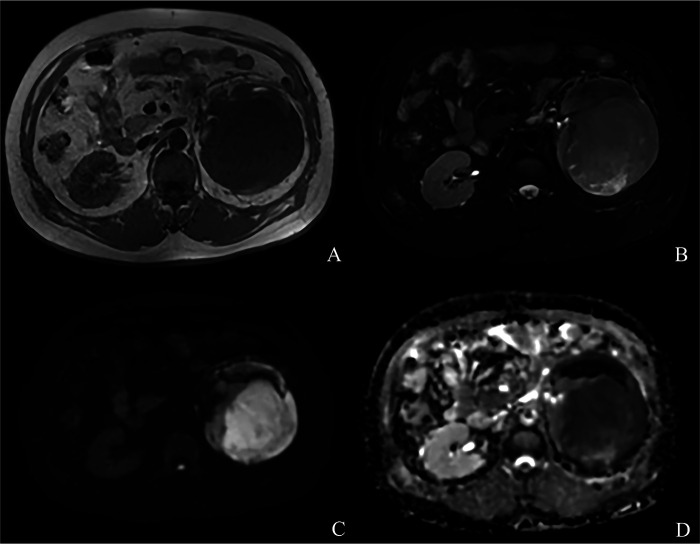
MRI of both kidneys: **(A)** slightly low signal on T1WI **(B)** slightly high signal on T2WI **(C)** high signal on DWI **(D)** ADC was decreased.

Three months prior to admission, a 53-year-old male patient developed intermittent left waist and abdominal pain without obvious cause. The pain was intermittent, with varying severity. His urinary flow was normal, and he had no frequent micturition, urgency, dysuria, hematuria, or fever. There were no other special discomforts. Upon hospital admission, a CT scan of both kidneys showed a possible malignant mass in the left kidney, approximately 13.3 cm × 11.6 cm in size. The density within was uneven, with CT values ranging from 25 to 58 HU. After enhancement, progressive enhancement was observed, with patchy slightly hypodense areas and fine vascular shadows inside (Figures 1A–D). Further MRI examination of both kidneys revealed a mass-like mixed signal lesion in the left kidney, with slightly low signal on T1WI (Figure 2A) and slightly high signal on T2WI (Figure 2B). The DWI showed high signal (Figure 2C), ADC was decreased (Figure 2D), and contrast-enhanced scan showed significant and uneven enhancement. Preoperative evaluations, including chest CT, abdominal ultrasound, and cardiac echocardiography, revealed no significant abnormalities.

**Figure 3:**
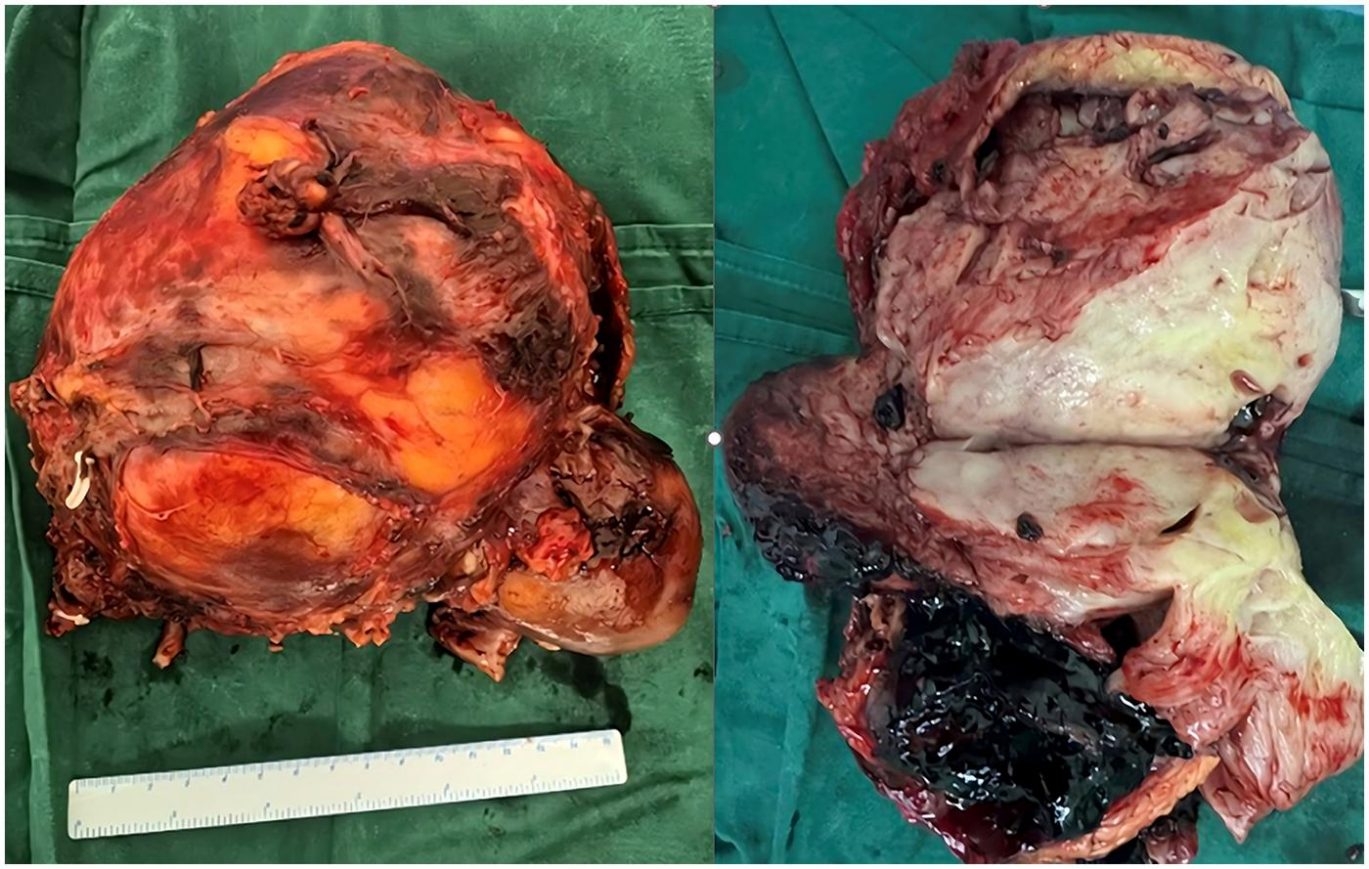
Gross examination of the specimen showed that the kidney was approximately 11.5 × 5.0 × 5.0 cm in size. The corticomedullary junction was clearly demarcated on the cut surface, with a cortical thickness of 0.6 cm. The renal pelvic mucosa was grayish-white and smooth. A tumor, measuring approximately 12.0 × 9.0 × 8.0 cm, was found in the perirenal fat capsule adjacent to the renal capsule. The cut surface of the tumor was grayish-white, soft, and fish-fleshed, with visible hemorrhage and necrosis.

This patient underwent radical resection of the left kidney tumor. During the operation, severe adhesions were observed around the kidney, the left kidney was fixed with poor mobility, and it was difficult to dissect the renal artery and renal vein. The tumor had abundant nutrient vessels around it. The originally planned laparoscopic surgery was converted to open surgery. Intraoperatively, the tumor tissue was severely adherent to the psoas major muscle, and no abnormalities were found during adrenal gland exploration (Figure 3).

**Figure 4:**
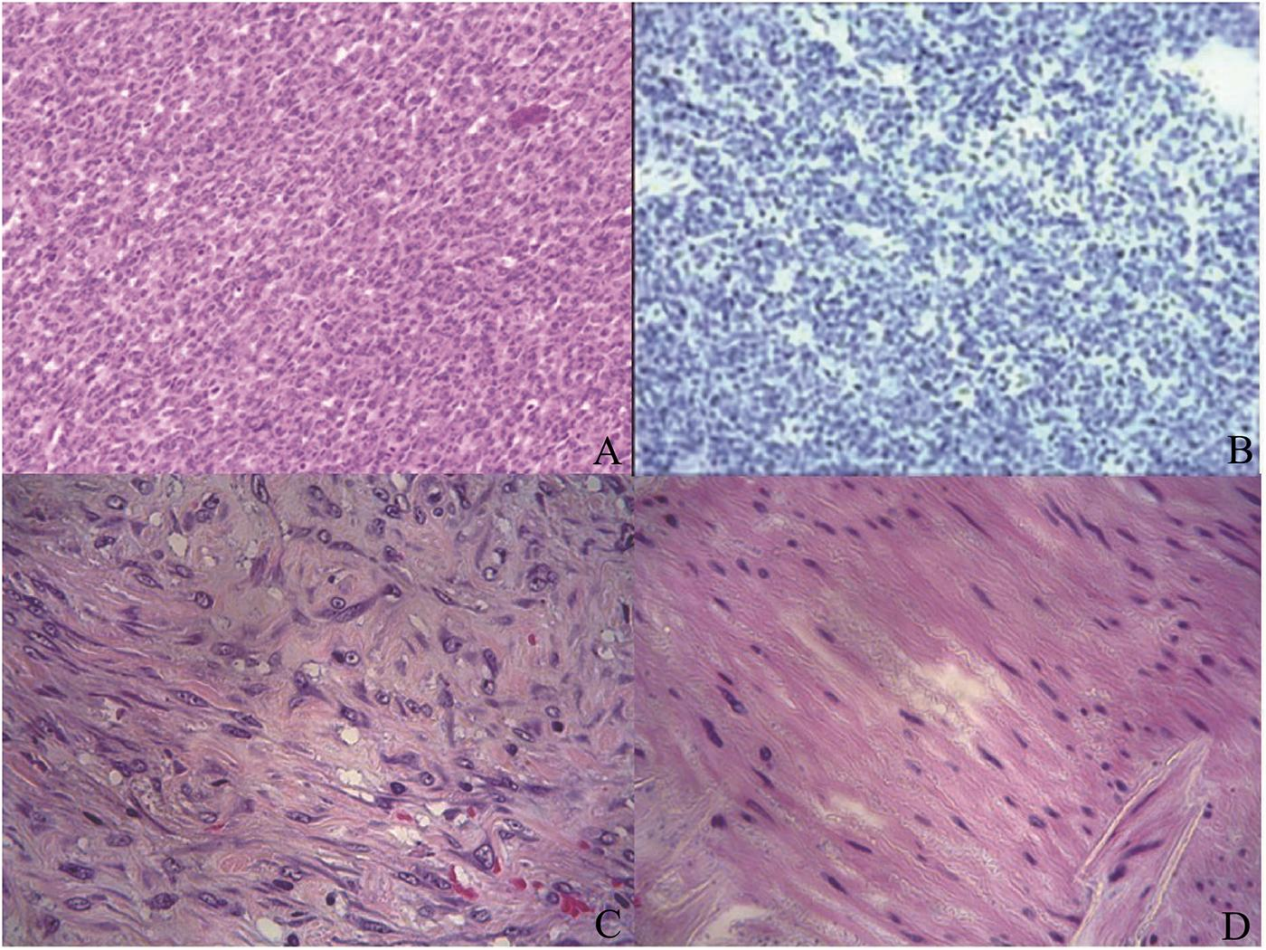
**(A)** abundant capillary formation was observed among closely arranged blue round tumor cells. The tumor cells were pleomorphic, round or oval in shape, with numerous mitotic figures. HE × 100. **(B)** PD-L1 × 200, TPS < 1%, CPS < 1. **(C,D)** The pathological sections reveal a prominent proliferation of spindle cells, with no evident epithelioid components observed.HE × 400.

The postoperative pathology revealed a high-grade mesenchymal malignant tumor with invasive growth. No definite lymphovascular invasion or perineural invasion is identified. The renal pelvis, adrenal gland, ureteral margin, and vascular margin are all free of tumor. No metastatic tumor is found in the renal hilar lymph nodes. Immunohistochemistry results were as follows: CK (partially positive), Vimentin (+), HMB45 (-), S-100 (-), CD34 (vascular +), Bcl-2 (+), EMA (-), Dog-1 (-), ERG (-), SMA (-), Ki-67 (30+) (Figure 4A). These findings suggested synovial sarcoma. We performed further genetic testing on the patient's tumor tissue specimen and peripheral blood using NGS combined with IHC. The results revealed the presence of an SS18-SSX2 fusion gene, thereby confirming the diagnosis of synovial sarcoma.

The patient received four cycles of chemotherapy with epirubicin combined with ifosfamide one month after surgery, which proceeded smoothly. No evidence of tumor recurrence or metastasis was found on re-examination after four cycles of chemotherapy (Table 1).

**Table 1:** Timeline of care.

Time point	Key events and findings
Preoperative	CT and MRI scans of both kidneys indicated a space-occupying lesion in the left kidney, raising suspicion for malignancy. Laparoscopic radical left nephrectomy was planned.
Surgery	Intraoperatively, severe perirenal adhesions with limited mobility were observed, making dissection of the renal artery and vein challenging. The tumor was noted to have abundant feeder vessels. Consequently, the procedure was converted from the planned laparoscopic approach to an open surgery.
Postoperative day 9	The postoperative pathology revealed a high-grade mesenchymal malignant tumor with invasive growth. Immunohistochemistry results were as follows: CK (partially positive), Vimentin (+), HMB45 (-), S-100 (-), CD34 (vascular +), Bcl-2 (+), EMA (-), Dog-1 (-), ERG (-), SMA (-), Ki-67 (30+) (Figure 4). These findings suggested synovial sarcoma.
Postoperative day 15	Genetic testing revealed an SS18-SSX2 fusion, confirming the diagnosis of synovial sarcoma.
One month postoperatively	The first cycle of chemotherapy with epirubicin (100 mg) and ifosfamide (2.5 g) was completed smoothly, and the patient exhibited no significant adverse reactions.
During chemotherapy cycles (administered every 21 days)	The patient received the second, third, and fourth cycles of chemotherapy (epirubicin 100 mg + ifosfamide 2.5 g per cycle). All treatments were well-tolerated without significant adverse reactions.
After completion of the four cycles of chemotherapy	Follow-up contrast-enhanced CT scans of the chest, upper abdomen, lower abdomen, and pelvis showed no definitive evidence of recurrence or metastasis.

## 3 Discussion

Synovial sarcoma is a relatively rare malignant tumor belonging to the category of undifferentiated soft tissue sarcomas (STS), accounting for 5%–10% of all STS cases (3). The incidence of synovial sarcoma is lower than that of other common STS subtypes, such as leiomyosarcoma, malignant fibrous histiocytoma, and liposarcoma (4). Synovial sarcoma was first reported in 1865, and it was named because it was described as a tumor adjacent to joints and its histological features were similar to those of developing synovium at that time. Although subsequent studies have shown that this tumor originates from mesenchymal cells and does not possess synovial characteristics, the name has been retained. At the same time, synovial sarcoma can also occurs in organs and tissues other than joints, such as the lungs (5), heart (6), prostate (7), and even the urethra (8). Renal synovial sarcoma, as in this case, is even rarer, accounting for less than 2% of all renal malignant tumors, with a slight male predominance (9). It was first reported by Argani P et al. in 2000 (10).

Histologically, synovial sarcoma is classified into monophasic synovial sarcoma (composed entirely of spindle cells), biphasic synovial sarcoma (containing both epithelial and spindle cells), and poorly differentiated synovial sarcoma, with the poorly differentiated type accounting for approximately 20% of cases and associated with a poorer prognosis (3, 11). In this case, the pathological sections reveal a prominent proliferation of spindle cells, with no evident epithelioid components observed, consistent with a diagnosis of monophasic synovial sarcoma (Figures 4C,D).

Studies have shown that chromosome translocation (t: 18; p11: q11) can be observed in almost all synovial sarcoma specimens. This translocation causes the SYT gene to fuse with the SSX1 or SSX2 genes on the X chromosome (12), forming a chimeric protein with transcriptional regulatory and nuclear localization functions (13, 14). The fusion gene SS18-SSX is the basis of the pathogenesis of synovial sarcoma (15), and genetic testing is the gold standard for its diagnosis. In this case report, the patient's genetic testing indicated a SS18-SSX2 fusion. Additionally, studies have confirmed that Bcl-2 is expressed in the vast majority of synovial sarcoma cases, and excessive Bcl-2 expression appears to be both an essential change in the pathogenesis of synovial sarcoma and a useful marker for differential diagnosis (11). Foo W C et al. detected TLE1 positivity in 60 of 73 synovial sarcoma cases, suggesting that TLE1 is a sensitive and highly specific marker for synovial sarcoma, which helps to distinguish it from lesions with similar histological features (16). Consequently, additional immunohistochemical staining was performed on the pathological specimen from this case, which revealed positive staining for TLE1. Most SS cases are significantly positive for Vimentin, while SMA, CK, CD34, CD31, HMB45, and S-100 are mostly negative (17). However, it should be noted that while immunohistochemical investigations are crucial, they may not always be conclusive, at least in one case, for diagnostic purposes (18). In clinical practice, a combination of immunohistochemistry and genetic testing is required for definitive diagnosis.

Renal synovial sarcoma has no specific clinical manifestations and is similar to common renal cell carcinoma. It often presents with abdominal pain, hematuria, and abdominal masses, with the lungs and liver are the most common sites of distant metastasis (9). At the same time, renal synovial sarcoma does not have typical imaging features. A previous case report showed that in preoperative CEUS, PRSS had the hyperenhancement feature of “ slow entry and fast exit”, and its special feature may have preoperative diagnostic value (19). Lv et al. summarized the enhanced CT findings of 5 patients with PRSS, indicating that all 5 cases exhibited the “fast-in and slow-out” enhancement pattern. In the present case, the enhanced CT of this patient also demonstrated the “fast-in and slow-out” imaging feature (20). In contrast, most renal cell carcinomas typically present with a “fast-in and fast-out” pattern. Enhanced CT may hold significant preliminary screening value in the differential diagnosis of renal cell carcinoma.

In this case, we further conducted genetic analysis and detected relevant gene mutations (Table 2), but the detected mutant genes did not have clinical therapeutic significance. The TMB-L of the detected TMB was 3.3 Muts/Mb, with a low load, and MSS microsatellite stability, which all suggest that this tumor may be insensitive to immune checkpoint inhibitors. At the same time, no harmful variations in HRR pathway-related genes or other core components of DDR pathway were detected, indicating that this patient may not be sensitive to PARP inhibitors. The PD-L1 test result showed that TPS was <1% and CPS was <1 (Figure 4B), which suggests that this patient may have low sensitivity to PD-L1 inhibitors and poor efficacy. Currently, there is no standard treatment guideline for primary renal synovial sarcoma, and surgical treatment is still the preferred option for PRSS, combined with postoperative chemotherapy. The most commonly used chemotherapy regimen is based on ifosfamide, combined with doxorubicin or epirubicin (9, 21). In this report, the patient received four cycles of chemotherapy with epirubicin combined with ifosfamide one month after surgery. No evidence of tumor recurrence or metastasis was found on re-examination after four cycles of chemotherapy. The patient reported no obvious discomfort or adverse reactions, and the patient will continue to be followed up. Given the rarity of primary renal synovial sarcoma, future progress will likely depend on integrating molecular diagnostics with translational platforms such as patient-derived primary cultures to enable functional testing and hypothesis-driven therapy selection (22). Meanwhile, soft tissue sarcomas show substantial biological heterogeneity, and immune/tumor microenvironment determinants are increasingly recognized as relevant for stratification and therapeutic development (23).

**Table 2:** The detected variant site.

gene	Transcript Number	Exon	Nucleotide Change	Amino Acid Change	Variant Type	Abundance/Copy Number
ERF	NM_006494.3	exon4	c.1365G > C	p.K455N	Missense Mutation	3.30%
ERCC2	NM_000400.3	exon11	c.1030C > G	p.L344V	Missense Mutation	5.90%
ERF	NM_006494.3	exon4	c.1140G > C	p.Q380H	Missense Mutation	3.10%
FOXO1	NM_002015.3	exon1	c.292G > C	p.A98P	Missense Mutation	4.70%
KMT2C	NM_170606.2	exon8	c.1013C > T	p.S338L	Missense Mutation	4.30%
NKX2-1	NM_003317.3	exon1	c.187G > C	p.A63P	Missense Mutation	4.20%
TBX3	NM_016569.3	exon8	c.1837G > C	p.A613P	Missense Mutation	3.30%
ZNF217	NM_006526.2	exon4	c.1829G > C	p.S610T	Missense Mutation	37.50%

## 4 Conclusions

Primary renal synovial sarcoma is a rare type of renal malignant tumor. Its clinical manifestations and imaging features lack specificity and are easily confused with common renal cell carcinoma. It is recommended to combine immunohistochemistry and SS18-SSX fusion gene testing after the operation to confirm the diagnosis. Radical surgical resection combined with epirubicin and ifosfamide chemotherapy is currently a safe and effective treatment option. This case can provide a reference for clinical diagnosis and treatment.

## Data Availability

The original contributions presented in the study are included in the article/Supplementary Material, further inquiries can be directed to the corresponding author.
